# Impact of Gliflozins on Right Heart Remodeling in Italian Patients with Type 2 Diabetes and Heart Failure: Results from the GLISCAR Real-World Study

**DOI:** 10.3390/ph18081200

**Published:** 2025-08-14

**Authors:** Erica Vetrano, Raffaele Galiero, Vittorio Simeon, Giuseppe Palmiero, Arturo Cesaro, Alfredo Caturano, Luca Rinaldi, Teresa Salvatore, Roberto Ruggiero, Maria Rosaria Di Palo, Celestino Sardu, Raffaele Marfella, Paolo Calabrò, Ferdinando Carlo Sasso

**Affiliations:** 1 Department of Advanced Medical and Surgical Sciences, University of Campania Luigi Vanvitelli, 80138 Naples, Italyraffaele.marfella@unicampania.it (R.M.); 2 Medical Statistics Unit, Department of Physical and Mental Health and Preventive Medicine, University of Campania Luigi Vanvitelli, Piazza Luigi Miraglia 2, 80138 Naples, Italy; 3 Department of Translational Medical Sciences, University of Campania Luigi Vanvitelli, 80131 Naples, Italy; 4 Division of Cardiology, A.O.R.N. “Sant’Anna & San Sebastiano”, 81100 Caserta, Italy; 5 Department of Human Sciences and Promotion of the Quality of Life, San Raffaele Roma Open University, 00166 Rome, Italy; 6 Department of Medicine and Health Science “Vincenzo Tiberio”, Università degli Studi del Molise, 86100 Campobasso, Italy; 7 Department of Precision Medicine, University of Campania Luigi Vanvitelli, 80138 Naples, Italy; 8 ASL Napoli 1 Centro, 80128 Napoli, Italy

**Keywords:** type 2 diabetes, sodium–glucose cotransporter 2 inhibitors, heart failure with reduced ejection fraction, real-world observational study, right ventricular function

## Abstract

**Aims**: The effect of sodium–glucose cotransporter 2 inhibitors (SGLT2is) in addition to optimal medical therapy (OMT) on right ventricular (RV) systolic function in patients with heart failure with reduced ejection fraction (HFrEF) is not well established. This study aimed to assess the impact of SGLT2is on RV function using advanced echocardiographic parameters in patients with HFrEF and type 2 diabetes (T2D). **Methods**: The real-world prospective, observational GLISCAR study enrolled 31 consecutive patients with T2D and HFrEF. All participants underwent clinical evaluation, laboratory testing, and comprehensive echocardiography at baseline and after 12 months of treatment with an SGLT2i. **Results**: After 12 months, statistically significant improvements in RV function were observed. Tricuspid annular plane systolic excursion (TAPSE) increased from 18.00 mm (SD ± 4.23; 95% confidence interval (CI): 16.51–19.49 mm) to 19.40 mm (SD ± 4.13; 95% CI: 17.95–20.85 mm) (*p* = 0.0346), and pulmonary artery systolic pressure (PASP) decreased from 35.23 mmHg (SD ± 14.61; 95% CI: 30.09–40.37 mm) to 30.89 mmHg (SD ± 7.77; 95% CI: 28.15–33.63 mm) (*p* < 0.001). These changes may suggest favorable RV remodeling and improved right ventricular–arterial coupling (RVAC). **Conclusions**: SGLT2i therapy was associated with improved RV function and RVAC in patients with HFrEF and T2D. While these findings are preliminary and drawn from a small, observational cohort, they support a potential role for SGLT2is in right heart remodeling. Further randomized, controlled studies are needed to confirm these effects and clarify their clinical implications.

## 1. Introduction

**Figure 1:**
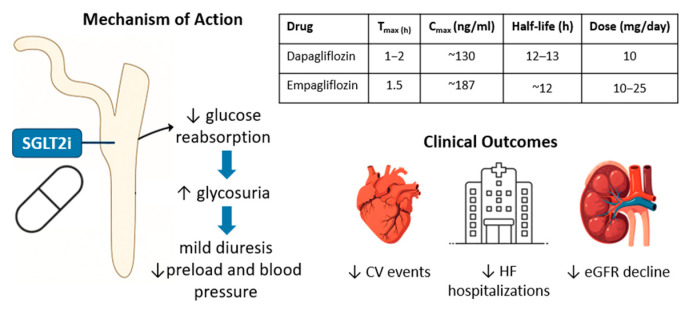
Pharmacokinetics, mechanism of action, and clinical benefits of dapagliflozin and empagliflozin in heart failure. SGLT2 inhibitors reduce glucose and sodium reabsorption in the proximal renal tubule, promoting glycosuria and mild osmotic diuresis. This mechanism contributes to cardiorenal protection, with no hypoglycemia risk when used as a monotherapy. The table summarizes pharmacokinetic parameters for dapagliflozin and empagliflozin. SGLT2i, Sodium–Glucose Cotransporter 2 inhibitor; CV, Cardiovascular; eGFR, estimated Glomerular Filtration Rate.

**Figure 2:**
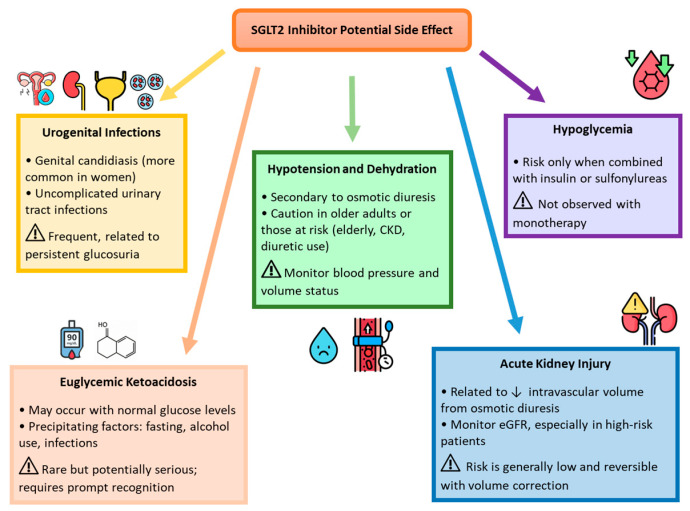
Potential adverse effects of sodium–glucose cotransporter 2 (SGLT2) inhibitors. Illustration summarizing the most common and clinically relevant adverse effects of SGLT2 inhibitor therapy, including urogenital infections, euglycemic ketoacidosis, hypotension and dehydration, hypoglycemia, and acute kidney injury. Risk statements emphasize frequency, precipitating factors, and monitoring recommendations. SGLT2, sodium–glucose cotransporter 2; CKD, Chronic Kidney Disease; eGFR, estimated Glomerular Filtration Rate.

In Western countries, approximately 500 million individuals are affected by type 2 diabetes (T2D), which is associated with a twofold increased risk of developing heart failure with reduced ejection fraction (HFrEF) compared to individuals without T2D. This elevated risk is primarily driven by hyperglycemia, insulin resistance, and systemic inflammation. HFrEF in patients with T2D remains a major contributor to global morbidity and mortality, associated with a higher risk of hospitalization (HR 4.73) and cardiovascular death (HR 2.45) [1,2]. These pathophysiological factors promote myocardial fibrosis, adverse cardiac remodeling, and endothelial dysfunction, thereby accelerating heart failure progression [3]. In recent years, cardiovascular risk reduction in patients with T2D has significantly improved with the advent of newer pharmacological agents, including sodium–glucose cotransporter 2 inhibitors (SGLT2is) and glucagon-like peptide-1 receptor agonists (GLP-1 RAs). GLP-1 RAs, such as semaglutide, have shown consistent benefits beyond glycemic control, including reductions in body weight, improvements in metabolic parameters, and favorable changes in body composition in individuals with T2D and obesity [4,5]. Treatment with semaglutide has also been associated with improvements in patient-reported quality of life and has demonstrated efficacy in both subcutaneous and oral formulations, broadening its applicability in clinical practice [6]. Despite their proven cardiovascular benefit in T2D, the role of GLP-1 RAs in heart failure management, particularly among non-diabetic patients, remains limited, and current guidelines do not recommend them as a first-line option for individuals with established heart failure [7,8]. On the other hand, SGLT2is have revolutionized the management of both T2D and HFrEF. Landmark studies, including EMPA-REG OUTCOME, CANVAS, and DAPA-HF, have demonstrated that SGLT2is not only improve glycemic control but also significantly reduce cardiovascular mortality and heart failure-related hospitalizations, independently of diabetes status [9,10,11]. Among the available agents, dapagliflozin and empagliflozin are the SGLT2 inhibitors currently approved for the treatment of heart failure according to European Society of Cardiology clinical practice guidelines [7]. Their pharmacokinetic properties, mechanisms of action, and established cardiorenal benefits are summarized in Figure 1. These benefits are attributed to pleiotropic mechanisms, including natriuresis, improved myocardial metabolism, the mitigation of oxidative stress, anti-inflammatory effects, and the attenuation of left ventricular (LV) systolic and diastolic dysfunction and fibrosis [10,11,12,13,14]. Although they are generally well tolerated, these agents may cause adverse effects such as urogenital infections, volume depletion, and, rarely, euglycemic ketoacidosis, warranting appropriate monitoring (Figure 2). Despite robust evidence for their impact on LV function, the effects of SGLT2is on right ventricular (RV) structure and function remain less well studied. The right ventricle, often under-recognized due to its complex anatomy and geometry and technical challenges in imaging, plays a crucial role in determining the prognosis of patients with heart failure, pulmonary hypertension, or ischemic heart disease [15,16,17]. In HFrEF, RV dysfunction is a powerful predictor of adverse outcomes and contributes to systemic congestion, exercise intolerance, and reduced quality of life [18]. Echocardiographic parameters such as tricuspid annular plane systolic excursion (TAPSE), pulmonary artery systolic pressure (PASP), and the TAPSE/PASP ratio are essential for assessing RV function and its interaction with pulmonary circulation [19]. TAPSE quantifies the longitudinal contraction of the RV, while PASP reflects the hemodynamic load imposed by pulmonary circulation. The TAPSE/PASP ratio, a marker of right ventricle–pulmonary artery coupling (RVAC), provides a comprehensive evaluation of RV function relative to its afterload and has emerged as a robust predictor of survival in a variety of cardiac conditions [20,21,22,23].

Previous findings have demonstrated that six months of SGLT2i therapy was associated with improvements in biventricular function, including early signs of favorable RV remodeling [24]. Building upon these observations and in accordance with the GLISCAR protocol, we extended the follow-up period to 12 months in order to evaluate the durability, progression, and potential stabilization of these cardiac effects over a longer period. The primary aim of this extended study was to confirm and consolidate the cardiovascular benefits observed at 6 months. The secondary aims were to explore the longitudinal trajectory of RV-specific echocardiographic parameters and to assess the sustained impact of SGLT2i therapy on cardiac remodeling, particularly in relation to right atrial function and RVAC.

## 2. Result

### 2.1. General and Clinical Characteristics of the Study Population at Baseline

At baseline, 31 individuals with HFrEF and T2D were enrolled, including 24 males (76.7%) and 7 females (23.3%). The mean age was 63.1 (SD ± 15.7) years, and there was a mean HbA1_c_ of 8.85 (SD ± 2.2). The median values of body weight and body mass index (BMI) were 89.0 kg [IQR 72.5–97.0] and 29.39 kg/m^2^ [IQR 25.7–32.7], respectively. Ischemic heart disease was the predominant etiology of HFrEF (73.0%), while the remaining cases were attributed to dilated cardiomyopathy. According to the New York Heart Association (NYHA) classification, 17 patients (54.9%) were class II, 13 (41.9%) were class III, and 1 (3.2%) was class IV. All patients were on beta-blockers at enrollment. Additional heart failure therapies included angiotensin receptor-neprilysin inhibitors (54.8%), ACE inhibitors (22.0%), angiotensin receptor blockers (12.9%), potassium-sparing diuretics (64.5%), and loop diuretics (67.7%). Other cardiovascular medications included ivabradine (6.4%), ranolazine (6.4%), digoxin (3.2%), calcium channel blockers (12.9%), and nitrates (3.2%). Antidiabetic treatments included metformin (45.1%) and insulin (25.8%). Antithrombotic therapy comprised acetylsalicylic acid (64.5%), P2Y12 inhibitors (61.2%), and oral anticoagulants (16.1%). Lipid-lowering agents included statins (67.6%), ezetimibe (51.6%), and PCSK9 inhibitors (19.3%). No patient had previously received SGLT2 inhibitor therapy. Regarding device therapy, 32.2% of patients had an implantable cardioverter-defibrillator (ICD) and 32.2% had received cardiac resynchronization therapy (CRT), while 6.4% had a pacemaker (PMK). All baseline characteristics are described in Table 1.

### 2.2. Changes in Clinical Characteristics and Blood Sample Findings

At the 12-month follow-up, changes were observed in both clinical and laboratory parameters compared to baseline (Table 2).

Among clinical characteristics, there were significant reductions in both body weight and BMI. Median body weight decreased from 89.0 kg [IQR: 72.5–97.0] to 86.5 kg [IQR: 67.75–92.75] (*p* < 0.001), and BMI declined from 29.39 kg/m^2^ [IQR: 25.69–32.71] to 29.00 kg/m^2^ [IQR: 24.25–33.00] (*p* < 0.001). Mean systolic and diastolic blood pressure values showed numerical increases from baseline (systolic blood pressure: 111.4 ± 15.98 mmHg to 119.0 ± 10.2 mmHg; diastolic blood pressure: 64.17 ± 10.51 mmHg to 70.66 ± 8.71 mmHg), although these changes did not reach statistical significance (*p* = 0.277 and *p* = 0.178, respectively).

Laboratory parameters demonstrated notable improvements. Mean HbA1c decreased significantly from 8.85 ± 2.20% to 6.72 ± 0.83% (*p* < 0.001), indicating improved glycemic control. Median NT-proBNP levels were significantly reduced from 1159.0 pg/mL [IQR: 892.5–2758.5] to 538.0 pg/mL [IQR: 328.25–2458.0] (*p* = 0.027), suggesting a favorable effect on heart failure status. Renal function remained stable over time. Mean serum creatinine increased slightly from 1.01 ± 0.31 mg/dL to 1.16 ± 0.41 mg/dL (*p* = 0.248), while median eGFR values showed a non-significant upward trend (63.0 mL/min/1.73 m^2^ [IQR: 52.5–85.0] to 63.5 mL/min/1.73 m^2^ [IQR: 46.5–76.5]; *p* = 0.206). Lipid parameters demonstrated minor, non-significant changes. Median LDL cholesterol declined modestly from 66.0 mg/dL [IQR: 40.0–91.0] to 62.0 mg/dL [IQR: 43.0–81.5] (*p* = 0.597), while HDL cholesterol showed a small increase (from 38.5 mg/dL [IQR: 31.5–54.0] to 40.0 mg/dL [IQR: 31.25–56.5]; *p* = 0.211). Median triglyceride levels also declined slightly (from 112.0 mg/dL [IQR: 87.5–147.5] to 111.0 mg/dL [IQR: 81.0–126.5]; *p* = 0.229), and total cholesterol remained virtually unchanged (*p* = 0.978). Hematologic parameters, including hemoglobin and hematocrit, increased slightly but without statistical significance (Hb: 12.81 ± 2.29 g/dL to 13.57 ± 1.92 g/dL, *p* = 0.298; HcT: 38.20 ± 4.60% to 40.88 ± 7.78%, *p* = 0.833).

All patients successfully completed the 12-month follow-up period while continuing SGLT2 inhibitor therapy. No major adverse events related to treatment were reported. Medication adherence was consistently high, as confirmed by regular clinical assessments and routine medication reviews. Throughout the study, none of the participants required dose adjustments or treatment discontinuation due to side effects or intolerance, further supporting the favorable tolerability of SGLT2is in this real-world cohort.

### 2.3. Assessment of Echocardiography in the Study Population: Time 0’ and 1’ (12 Months Follow-Up)

All participants underwent transthoracic echocardiography at baseline and after 12 months of follow-up. Over this period, several changes were observed in both left and right heart parameters, reflecting favorable cardiac remodeling.

LV structure and function improved notably. There were significant reductions in left ventricular end-diastolic diameter (LVEDD): (61.29 ± 7.93 mm to 58.34 ± 7.03 mm; *p* = 0.014) and left ventricular end-systolic diameter (LVESD) (49.61 ± 10.11 mm to 45.03 ± 9.01 mm; *p* = 0.003). Interventricular septal thickness (IVSD) showed a modest but significant increase (from 11.00 mm [IQR: 9.50–12.00] to 11.00 mm [IQR: 10.00–12.00]; *p* = 0.006). Correspondingly, both left ventricular end-diastolic volume (LVEDV) (183.52 ± 59.00 mL to 171.00 ± 57.37 mL; *p* = 0.009) and left ventricular end-systolic volume (LVESV) (123.45 ± 46.96 mL to 106.10 ± 44.02 mL; *p* = 0.007) were significantly reduced. Although the increase in left ventricular ejection fraction (LVEF) from 33.06 ± 5.36% to 38.71 ± 7.21% did not reach statistical significance (*p* = 0.075), a clear upward trend was observed. A significant improvement was also recorded in global longitudinal strain (GLS), with median values improving from –8.40% [IQR: –10.30 to –7.80] to –9.70% [IQR: –8.05 to –12.05]; (*p* = 0.009). Additionally, the left atrial diameter (LAD) decreased from 46.00 mm [IQR: 42.50–50.00] to 43.00 mm [IQR: 41.00–49.00]; (*p* = 0.026), suggesting reduced left atrial pressure or volume load.

Right heart function also showed meaningful improvements. Right atrial function improved significantly, with the right atrial emptying index (RAEI) increasing from 78.22 ± 51.69 mL to 92.00 ± 48.40 mL (*p* = 0.003), and total right atrium emptying fraction (TRAEF) increasing from 40.00 ± 15.16% to 45.05 ± 13.00% (*p* = 0.012).

Regarding RV performance, TAPSE improved from 18.00 ± 4.23 mm (95% confidential interval (CI): 16.51–19.49 mm) to 19.40 ± 4.13 mm (95% CI: 17.95–20.85 mm) (*p* = 0.035), and the RV fractional area change (FAC) increased from 39.20 ± 7.17% (95% CI: 36.68–41.72%) to 40.03 ± 7.13% (95% CI: 37.52–42.54%) (*p* < 0.001). PASP significantly declined from 35.23 ± 14.61 mmHg (95% CI: 30.09–40.37 mm) to 30.89 ± 7.77 mmHg (95% CI: 28.15–33.63 mm) (*p* < 0.001), indicating reduced pulmonary pressure. Other RV dimensions, including right ventricular diameter (RVD) 1 and RVD2, showed non-significant increasing trends, whereas RVD3 showed a slight but statistically significant reduction (from 75.84 ± 10.86 mm to 75.37 ± 8.83 mm; *p* = 0.001). The TAPSE/PASP ratio increased from 0.58 ± 0.24 to 0.69 ± 0.23 (*p* = 0.388), although the change was not statistically significant.

A complete summary of all echocardiographic parameters is provided in Table 3.

To better understand the observed differences between baseline and 12-month follow-up, and to identify potential modifiers of the changes in echocardiographic parameters over time, a detailed landmark analysis was conducted. This analysis focused on the delta (Δ) values (i.e., the difference between baseline and 12-month follow-up findings) and evaluated whether these changes were influenced by baseline values (regression toward the mean) and other factors, including age, sex, and BMI. From this analysis, only LVEF demonstrated a significant interaction with time and sex (Sex: *p* = 0.0011; Time: *p* < 0.0001; Sex x Time interaction *p* = 0.0097), as illustrated in Figure S1 of the Supplementary Materials. At baseline, LVEF values were comparable between males and females; however, after 12 months, females showed a greater improvement in LVEF compared to males, suggesting a sex-specific response to the treatment. Further details are provided in the Supplementary Materials (Figures S2–S13).

## 3. Discussion

In recent years, SGLT2is have redefined the therapeutic landscape of heart failure (HF). Originally developed as antihyperglycemic agents for T2D, these drugs have demonstrated clinically relevant cardioprotective effects, extending their clinical indications well beyond glycemic control [9,10,25,26]. Consequently, contemporary guidelines strongly endorse their use across the full spectrum of HF phenotypes, including HFrEF, heart failure with preserved ejection fraction (HFpEF), and heart failure with mildly reduced ejection fraction (HFmrEF) [27,28]. This recommendation is grounded in compelling evidence demonstrating the beneficial impact of SGLT2is on both LV and RV function, with improvements in ejection fraction, diastolic function, and myocardial remodeling [17,29]. While prior research has primarily focused on LV outcomes, the pivotal role of RV function in HF prognosis is increasingly recognized. RV dysfunction is associated with worse clinical outcomes, including increased morbidity, mortality, and diminished quality of life [30,31]. To address this knowledge gap, the current extension of the GLISCAR study conducted a comprehensive assessment of the effects of SGLT2is on RV function and its dynamic interaction with the pulmonary vasculature. The observed improvements in RV function are likely multifactorial. SGLT2is reduce pulmonary congestion, promote diuresis, and may improve pulmonary vascular compliance [32]. These mechanisms align with Bernoulli’s principle, wherein a reduction in pulmonary vascular resistance and RV filling pressure facilitates more efficient pulmonary circulation, ultimately enhancing RV function and RVAC [33]. However, as invasive hemodynamic assessments were not performed, the extent to which these changes reflect direct myocardial effects versus volume-mediated adaptations remains uncertain. Given the known diuretic and natriuretic properties of SGLT2is, it is plausible that reduced preload and afterload contributed to the observed improvements in right heart function. Clarifying whether these changes represent true myocardial remodeling or functional adaptation will require studies employing load-independent imaging techniques and direct hemodynamic measurements [34].

Among echocardiographic markers, a statistically significant increase in TAPSE, a key marker of RV longitudinal contractility, was observed. Additionally, a marked reduction in PASP indicated a decrease in RV afterload [35,36]. Quantitatively, TAPSE increased by approximately 1.4 mm, while PASP declined by over 4 mmHg, changes that are both statistically and clinically meaningful [20]. Improvements in FAC further support the enhancement of RV systolic function. Although the increase in LVEF did not reach statistical significance (*p* = 0.0754), this trend may reflect a meaningful clinical improvement, particularly among female patients.

At the molecular level, SGLT2is may improve myocardial efficiency by promoting ketone body oxidation, a more energy-efficient substrate compared to glucose [37]. Additionally, SGLT2is may attenuate oxidative stress, modulate intracellular calcium handling, and reduce myocardial fibrosis, all mechanisms contributing to improved compliance and contractility [38,39]. The observed reductions in pro-BNP and HbA1c levels further support the systemic effects of SGLT2is on myocardial workload and metabolic status, while the rise in eGFR aligns with their known nephroprotective actions [40].

A particularly intriguing aspect of this study is the identification of a sex-specific therapeutic response. Female patients exhibited a more pronounced improvement in LVEF compared to their male counterparts, suggesting a potentially amplified benefit of SGLT2is in women. This phenomenon may be attributed to several biological and metabolic factors. Estrogen is known to exert cardioprotective effects by modulating myocardial metabolism and reducing oxidative stress [41]. Moreover, women have a greater contractile reserve and are generally more responsive to pharmacologic therapies, including those targeting ketone metabolism. Differences in neurohormonal regulation, such as lower sympathetic tone and enhanced parasympathetic activity in women, may further augment the response to SGLT2is [42,43,44].

### 3.1. Perspective for Clinical Practice

Based on our results, SGLT2is may contribute to improve dynamic RV function. Patients with right-sided heart dysfunction often present with widespread edema, impaired exercise capacity, and abnormal hemodynamic responses to physical activity [45]. Clinically, the reduction in right-sided filling pressures may lead to improvements in symptoms, physical function, and overall quality of life [46]. In line with these observations, previous studies have reported beneficial effects of gliflozins on exercise tolerance and performance, including an increased 6 min walk distance [46,47]. Furthermore, recent clinical trials have demonstrated that SGLT2 inhibitors improve patient-reported outcomes, including health-related quality of life, symptom burden, and daily activity levels in patients with HFrEF [10,11,48]. These benefits are believed to result not only from enhanced ventricular function but also from reduced congestion, improved renal function, and greater metabolic efficiency [10,11,48]. In real-world settings, such multidimensional benefits may translate into better treatment adherence and reduced healthcare utilization, reinforcing the role of SGLT2is as a cornerstone of heart failure management. Therefore, to better evaluate treatment efficacy, it is clinically relevant for physicians to assess right heart function and its manifestations, particularly in patients treated with gliflozins who show limited improvements in left ventricular parameters or who have heart failure with HFpEF.

### 3.2. Limitations

Despite these promising findings, several limitations must be acknowledged. First, the non-controlled, observational design limits the ability to draw causal inferences and prevents the definitive attribution of the observed effects solely to SGLT2 inhibitor therapy. However, the prospective nature of the study and the stable clinical status of participants provided a solid framework for within-subject comparisons over time. While these preliminary results are not definitive, they offer valuable real-world insights and underscore the need for confirmation in future randomized controlled trials. Second, the small sample size and absence of an a priori power calculation may reduce statistical power and increase the risk of type II error. Notably, the initial 6-month evaluation in the same cohort (n = 31) demonstrated statistically significant improvements in both right and left ventricular function, including key parameters such as TAPSE and PASP. These findings suggested a clinically meaningful treatment effect and supported the extension of follow-up to 12 months. Given the real-world, prospective nature of the study and the absence of prior data specifically addressing RV remodeling in this population, the sample size was determined based on recruitment feasibility and the promising early echocardiographic response. This limitation, while acknowledged, is consistent with the exploratory objectives of the study and constrains its generalizability. Third, the absence of a control group precludes definitive attribution of observed effects to SGLT2i therapy. Although patients were on stable optimal medical therapy prior to SGLT2i initiation, the lack of a comparison arm or run-in period limits internal validity.

Future studies should aim to address these gaps by incorporating larger, more diverse patient populations, utilizing multicenter designs, and including control groups or within-subject comparators. Advanced imaging modalities and biomarker profiling will be crucial in elucidating the mechanisms of action and identifying responders. Furthermore, the potential for sex-specific treatment strategies should be explored in randomized, adequately powered trials to refine personalized approaches to heart failure management. Additionally, comparative studies assessing other cardiometabolic therapies, such as GLP-1 receptor agonists, may provide further insight into their potential role in right heart remodeling and quality of life improvements in this patient population.

## 4. Materials and Methods

### 4.1. Study Population

The GLISCAR study is a real-world, multicenter, prospective, observational cohort study that enrolled 31 consecutive individuals with T2D and chronic HFrEF, defined as a left ventricular ejection fraction (LVEF) ≤ 40%. The study was non-randomized and lacked a control group, reflecting real-world clinical practice. Participants were recruited between February 2021 and June 2022 from the Department of Translational Medical Sciences and the Division of Cardiology at A.O.R.N. “Azienda Ospedaliera dei Colli,” and A.O.R.N. “Sant’Anna & San Sebastiano,” Caserta, both affiliated with the University of Campania “Luigi Vanvitelli,” Naples, Italy. Patients with severe RV dysfunction, advanced pulmonary hypertension (PASP > 60 mmHg), were excluded to ensure a clinically homogeneous cohort and to focus the analysis on mild-to-moderate stages of RV remodeling, stages that are more likely to respond to pharmacological intervention. In individuals with advanced RV dysfunction, the therapeutic response is often limited due to irreversible structural changes; thus, their inclusion could have reduced the study’s sensitivity in detecting clinically meaningful effects of SGLT2 inhibition. Additionally, patients with renal impairment (eGFR < 30 mL/min/1.73 m^2^), clinically relevant anemia, or active malignancies were excluded from enrollment to minimize confounding factors and ensure patient safety.

**Figure 3:**
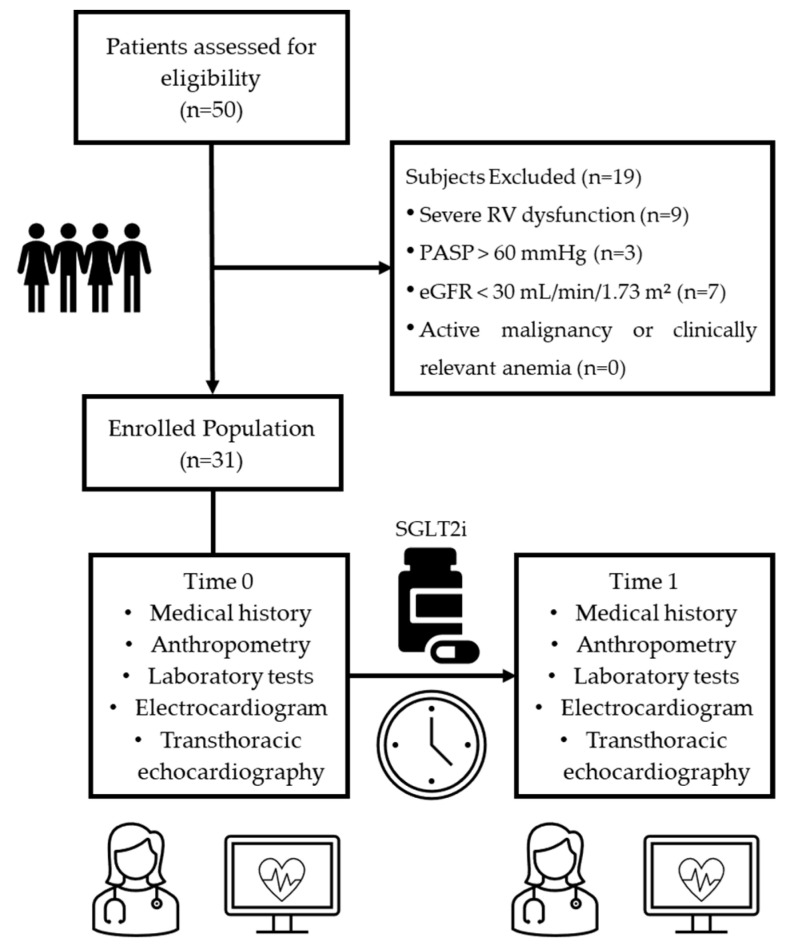
Study design and timeline. RV, Right Ventricular; PASP, Pulmonary Artery Systolic Pressure; eGFR, estimated Glomerular Filtration Rate; SGLT2i, Sodium–Glucose Cotransporter 2 inhibitor.

Patients were enrolled after achieving clinical stabilization on optimal medical therapy (OMT) to minimize hemodynamic variability prior to SGLT2i initiation. Patients received either empagliflozin (n = 21) or dapagliflozin (n = 10), both at the standard dose of 10 mg once daily, based on clinical judgment and drug availability. The decision to adopt a 12-month follow-up period was based on the need to evaluate not only the early effects but also the durability and progression of SGLT2i-related changes in cardiac structure and function over a clinically meaningful timeframe. This duration aligns with standard practice in heart failure monitoring and with the GLISCAR study protocol, which was designed to explore the potential for sustained or stabilizing effects on right heart remodeling. A 12-month period also allows for the detection of delayed or progressive treatment responses that may not be evident in shorter observational windows. The study design is summarized in Figure 3.

This study was conducted and reported in accordance with the STROBE (Strengthening the Reporting of Observational Studies in Epidemiology) guidelines for observational studies and adheres to the EQUATOR Network recommendations.

The study was approved by the Ethics Committee of the University of Campania “Luigi Vanvitelli”—AOU “Luigi Vanvitelli”—AORN “Ospedale dei Colli” (Approval ID: 2020/27967; date: 19 November 2020). All participants provided written informed consent prior to enrollment. The study was conducted in accordance with the principles of Good Clinical Practice and the Declaration of Helsinki (including its most recent amendments).

### 4.2. Clinical Assessment

Clinical and diagnostic data were collected at baseline and after 12 months of follow-up. Demographic information included age, sex, height, and body weight, from which the body mass index (BMI) was calculated. A detailed clinical history was obtained for each subject, including comorbidities such as hypertension, atrial fibrillation, and chronic kidney disease, as well as diabetes-related complications. Data on smoking status, prior cardiovascular events, and device implantation (such as ICD or CRT) were also recorded. Pharmacological treatments were systematically reviewed, encompassing heart failure medications (beta-blockers angiotensin-converting enzyme [ACE] inhibitors, angiotensin receptor blockers or neprilysin inhibitors, mineralocorticoid receptor antagonists, and diuretics), antidiabetic agents (e.g., metformin, insulin, dipeptidyl peptidase-4 [DPP-4] inhibitors), and other cardiovascular therapies such as lipid-lowering agents and antithrombotic drugs. Clinical assessments were complemented by resting 12-lead electrocardiograms and comprehensive transthoracic echocardiograms, performed under standardized conditions at baseline and at the end of the 12-month treatment period.

### 4.3. Laboratory Assessment

Laboratory venous blood samples were collected after overnight fasting at both baseline and follow-up visits. The biochemical profile included serum creatinine, fasting plasma glucose, glycated hemoglobin (HbA1c), total cholesterol, LDL cholesterol, HDL cholesterol, triglycerides, hematocrit, and N-terminal pro-B-type natriuretic peptide (NT-proBNP). Renal function was assessed using the estimated glomerular filtration rate (eGFR), calculated via the CKD-EPI formula [49].

### 4.4. Echocardiography

Transthoracic 2D echocardiography was performed for all subjects enrolled in the study, through an ultrasound system (Vivid E9, GE Healthcare, Milwau-kee, WI, USA) equipped with a 3.5 MHz transducer [50]. LV volumes were evaluated using Simpson’s biplane method and indexed to body surface area, and then LVEF was calculated as recommended. E wave, A wave, the E/A ratio, and E-deceleration time (DecT) were measured using pulsed Doppler from the four-chamber view. Tricuspid regurgitant jet velocities, inferior vena cava diameters, and respiratory variations were used to determine PASP. Tricuspid annular longitudinal excursions in an apical four-chamber view with M-mode modality were evaluated to obtain TAPSE. As a consequence, the TAPSE/PASP ratio has been calculated. The basal cavity RVD was measured in the basal one-third of the right ventricle on the 4-chamber view. FAC was obtained through the following formula: (RV diastolic area—RV systolic area)/RV diastolic area 100%.

Speckle tracking analysis was performed using dedicated software to obtain peak systolic longitudinal strain and 2D Speckle-tracking (2D-ST) strain measurements. According to guidelines, GLS was calculated by the average of regional LS values [51].

Left ventricular diameters have been measured from M-mode and 2-dimensional echocardiography (2DE). Particularly, LVEDD and LVEDV have been evaluated when the ventricle was largest, shortly before the walls started to move inward. The region in which the ventricular cavity was smallest was obtained to describe LVESD and volume (LVESV). IVSD was measured at end-diastole in the parasternal long axis. LA diameter and volumes were measured before mitral valve opening (LAVmax) and indexed for body surface area and expressed as an LAVI. Right atrium area (RAA) and volume (RAV) were measured before tricuspid valve opening and tracing the line from the plane of the tricuspid annulus along the superior and anterolateral walls of the RA and the interatrial septum (IAS). TRAEF was obtained through the following formula: maximum volume—minimum volume/maximum volume.

All echocardiographic exams were performed and analyzed by the same operator at each center to minimize interobserver variability.

### 4.5. Statistical Analysis

The Shapiro–Wilk test was used to verify the distribution of continuous variables. Consequently, these latter variables were expressed as median data and interquartile range [IQR] or mean data and standard deviation (SD). Categorical variables were shown as frequencies in absolute and relative percentage values. Population data have been evaluated at two different times (Time 0 at baseline and Time 1 at 12 months). To observe the difference between the two times, an overall *p* value was calculated (either a paired T-test or Wilcoxon matched-pairs Mann–Whitney for continuous data, depending on their distribution; McNemar’s test for categorical data).

After that, a delta (Δ) variable representing the change between baseline and 12-month values was calculated and used as a dependent variable in a linear model to assess the role of other clinical and demographic variables, such as age, gender, and BMI, adjusting for the baseline variable. So, a model was built to verify whether the difference between the two periods was influenced by the echocardiographic variable at baseline—the regression effect towards the mean—and subsequently, by the variables of age, sex, and BMI. In addition, the interaction effect has been evaluated in a repeated measures model to see if the drug efficacy over time is different in the different categories. All procedures have been conducted through Stata 18 software (StataCorp LLC, College Station, TX, USA; Stata Statistical Software: Release 18, 2023).). Given the exploratory nature of this study and the absence of prior data specifically addressing right heart remodeling in patients with HFrEF and T2D treated with SGLT2 inhibitors, no formal a priori sample size calculation was performed. Instead, the sample size was determined based on recruitment feasibility and is consistent with previous pilot studies conducted in similar clinical settings.

## 5. Conclusions

In conclusion, the extended GLISCAR study suggests that SGLT2 inhibitors may exert favorable effects on both left ventricular and RV function, and on pulmonary hemodynamics, in patients with HFrEF and T2D. These findings support the emerging evidence on the cardioprotective properties of SGLT2is, particularly regarding myocardial remodeling and right heart performance, areas that are often underrepresented in heart failure research. The observed echocardiographic improvements may translate into better exercise tolerance and quality of life, reinforcing the clinical value of assessing the RV function in this population. While the study’s observational design and modest sample size limit causal interpretation and generalizability, the real-world perspective and consistency with major clinical trial outcomes provide meaningful insights for clinical practice. Future studies should investigate the comparative effects of other cardiometabolic agents, such as GLP-1 receptor agonists, and further define the role of right heart remodeling as a therapeutic target in heart failure management.

## Figures and Tables

**Table 1:** Baseline clinical characteristics of the study sample.

Variable	Overall (n = 31)
Age, yrs, mean (SD)	63.10 (15.71)
Sex, n (%)	
Male	24 (76.7)
Female	7 (23.3)
Height, mean (SD)	170.55 (9.30)
NYHA Class, n (%)	12 (19.0)
I	-
II	17 (54.9)
III	13 (41.9)
IV	1 (3.2)
Arterial Hypertension, n (%)	28 (93.3)
Duration of Heart Failure, yrs, median [IQR]	9.5 (8.2–11.5)
Ischemic etiology, n (%)	22 (73.3)
Dilated cardiomyopathy, n (%)	9 (26.7)
Atrial fibrillation, n (%)	9 (30.0)
Smoking, n (%)	9 (30.0)
Retinopathy, n (%)	5 (16.7)
Neuropathy, n (%)	4 (13.3)
CKD, n (%)	9 (30.0)
Beta-blockers, n (%)	31 (100)
ACE-i, n (%)	7 (22)
Sartans, n (%)	10 (32)
Nepryl-i, n (%)	21 (68)
Anti-Mineralcorticoids, n (%)	24 (77)
Diuretics, n (%)	26 (84)
Ivabradine, n (%)	3 (10)
Ranolazine, n (%)	2 (6)
Ca^2+^-Antagonists, n (%)	6 (20)
Digoxin, n (%)	3 (10)
Nitrates, n (%)	1 (3)
Metformin, n (%)	9 (30)
Insulin, n (%)	6 (19)
Acetylsalicylic acid, n (%)	22 (70)
P2Y12-i, n (%)	22 (70)
Oral anticoagulant, n (%)	5 (16.1)
Ezetimibe, n (%)	18 (58)
Statins, n (%)	25 (80)
PCSK9-i, n (%)	6 (19.3)

SD, Standard Deviation; IQR, Interquartile Range BMI, Body Mass Index; NYHA, New York Heart Association; CKD, Chronic Kidney Disease, ACE-I, Angiotensin-Converting Enzyme Inhibitors; Nepryl-I, Neprylisin inhibitors; P2Y12-I, P2Y12 inhibitors; PCSK9-i, Proprotein Convertase Subtilisin/Kexin Type 9 inhibitors.

**Table 2:** Comparison of baseline and follow-up anthropometrical and laboratory data of the study sample.

Progression Time			
Variable	Time 0	Time 1 (=12 Months)	*p*
Weight, median [IQR]	89.00 [72.50–97.00]	86.50 [67.75–92.75]	<0.001
BMI, median [IQR]	29.39 [25.69–32.71]	29.00 [24.25–33.00]	<0.001
SBP, mean (SD)	111.40 (15.98)	119 (10.20)	0.277
DBP, mean (SD)	64.17 (10.51)	70.66 (8.71)	0.178
Hb, mean (SD)	12.81 (2.29)	13.57 (1.92)	0.298
HcT, mean (SD)	38.20 (4.60)	40.88 (7.78)	0.833
HbA1c, mean (SD)	8.85 (2.20)	6.72 (0.83)	<0.001
Creatinine, mean (SD)	1.01 (0.31)	1.16 (0.41)	0.248
eGFR, median [IQR]	63.00 [52.50–85.00]	63.5 [46.5–76.5]	0.206
Total cholesterol, median [IQR]	128.00 [103.25–174.75]	130.00 [111.00–162.00]	0.978
LDL cholesterol, median [IQR]	66.00 [40.00–91.00]	62.00 [43.0–81.5]	0.597
HDL cholesterol, median [IQR]	38.50 [31.50–54.00]	40.00 [31.25–56.50]	0.211
Triglyceride, median [IQR]	112.00 [87.50–147.50]	111.00 [81.00–126.50]	0.229
NT Pro-BNP, median [IQR]	1159.00 [892.50–2758.50]	538.00 [328.25–2458.00]	0.027

IQR, Interquartile Range; SD, Standard Deviation; BMI, Body Mass Index; SBP, Systolic Blood Pressure; DBP, Diastolic Blood Pressure; Hb, Hemoglobin; HcT, Hematocrit; Hb1A_c_, Glycated hemoglobin; eGFR, Estimated Glomerular Filtration Rate; NT Pro-BNP, N-Terminal Pro B-Type Natriuretic Peptide.

**Table 3:** Echocardiographic findings at baseline (time 0) and 12-month (time 1) follow-up.

Progression Time			
Variable	Time 0	Time 1 (=12 Months)	*p*
LVEDD (mm), mean (SD)	61.29 (7.93)	58.34 (7.03)	0.014
LVESD (mm), mean (SD)	49.61 (10.11)	45.03 (9.01)	0.003
IVSD (mm), median [IQR]	11.00 [9.50–12.00]	11.00 [10.00–12.00]	0.006
LVEDV (ml), mean (SD)	183.52 (59.00)	171 (57.37)	0.009
LVESV (ml), mean (SD)	123.45 (46.96)	106.10 (44.02)	0.007
LVEF (%), mean (SD)	33.06 (5.36)	38.71 (7.21)	0.075
GLS (%), median [IQR]	−8.40 [−10.30–−7.80]	−9.70 [−8.05–−12.05]	0.009
LAD (mm), median [IQR]	46.00 [42.50–50.00]	43.00 [41.00–49.00]	0.026
RAA (cm^2^), mean (SD)	17.12 (3.61)	16.73 (3.37)	0.100
RAEI (ml), mean (SD)	78.22 (51.69)	92.00 (48.40)	0.003
TRAEF (%), mean (SD)	40 (15.16)	45.05 (13.00)	0.012
RVD1 (mm), mean (SD)	39.55 (5.43)	40.00 (5.39)	0.069
RVD2 (mm), mean (SD)	30.87 (5.77)	32.21 (5.90)	0.363
RVD3 (mm), mean (SD)	75.84 (10.86)	75.37 (8.83)	0.001
TAPSE (mm), mean (SD)	18.00 (4.23)	19.40 (4.13)	0.035
FAC (%), mean (SD)	39.20 (7.17)	40.03 (7.13)	<0.001
PASP (mmHg), mean (SD)	35.23 (14.61)	30.89 (7.77)	<0.001
TAPSE/PAPs ratio, mean (SD)	0.58 (0.24)	0.69 (0.23)	0.388

SD, Standard Deviation; IQR, Interquartile Range; LVEDD, Left Ventricular End-Diastolic Diameter; LVESD, Left Ventricular End-Systolic Diameter; IVSD, Interventricular Septum Diameter; LVEDV, Left Ventricular End-Diastolic Volume; LVESV, Left Ventricular End-Systolic Volume; LVEF, Left Ventricular Ejection Fraction; GLS, Global Longitudinal Strain; LAD, Left Atrial Dimension; RAA, Right Atrial Area; RAEI, right atrial emptying index; TRAEF, Total right atrium emptying fraction; RVD1, Right Ventricular Diameter 1; RVD2 Right Ventricular Diameter 2; RVD3, Right Ventricular Diameter 3; TAPSE, Tricuspid Annular Plane Systolic Excursion; FAC; Fractional Area Shortening; PASP; Pulmonary Artery Systolic Pressure.

## Data Availability

The original contributions presented in the study are included in the article and Supplementary Materials; further inquiries can be directed to the corresponding authors.

## Supplementary Materials

The following supporting information can be downloaded at: https://www.mdpi.com/article/10.3390/ph18081200/s1, Figure S1. Left Ventricular End-Diastolic Diameter (LVEDD); Figure S2. Left Ventricular End-Systolic Diameter (LVESD); Figure S3. Interventricular Septum Diameter (IVSD); Figure S4. Left Ventricular End-Diastolic Volume (LVEDV); Figure S5. Left Ventricular End-Systolic Volume (LVESV); Figure S6. Global Longitudinal Strain (GLS); Figure S7. Right Atrial Area (RAA); Figure S8. Right atrial emptying index (RAEI); Figure S9. Total right atrium emptying fraction (TRAEF); Figure S10. Right Ventricular Diameter 3 (RVD3); Figure S11. Tricuspid Annular Plane Systolic Excursion (TAPSE); Figure S12. Fractional Area Shortening (FAC); Figure S13. Pulmonary Artery Systolic Pressure (PASP).

## References

[1] Palazzuoli A., Iacoviello M. (2023). Diabetes Leading to Heart Failure and Heart Failure Leading to Diabetes: Epidemiological and Clinical Evidence. Heart Fail. Rev..

[2] Bell D.S.H., Goncalves E. (2019). Heart Failure in the Patient with Diabetes: Epidemiology, Aetiology, Prognosis, Therapy and the Effect of Glucose-Lowering Medications. Diabetes Obes. Metab..

[3] Zhang H., Dhalla N.S. (2024). The Role of Pro-Inflammatory Cytokines in the Pathogenesis of Cardiovascular Disease. Int. J. Mol. Sci..

[4] Pantanetti P., Cangelosi G., Alberti S., Di Marco S., Michetti G., Cerasoli G., Di Giacinti M., Coacci S., Francucci N., Petrelli F. (2024). Changes in Body Weight and Composition, Metabolic Parameters, and Quality of Life in Patients with Type 2 Diabetes Treated with Subcutaneous Semaglutide in Real-World Clinical Practice. Front. Endocrinol..

[5] Rodríguez Jiménez B., Rodríguez de Vera Gómez P., Belmonte Lomas S., Mesa Díaz Á.M., Caballero Mateos I., Galán I., Morales Portillo C., Martínez-Brocca M.A. (2024). Transforming Body Composition with Semaglutide in Adults with Obesity and Type 2 Diabetes Mellitus. Front. Endocrinol..

[6] Meier J.J. (2021). Efficacy of Semaglutide in a Subcutaneous and an Oral Formulation. Front. Endocrinol..

[7] McDonagh T.A., Metra M., Adamo M., Gardner R.S., Baumbach A., Böhm M., Burri H., Butler J., Čelutkienė J., Chioncel O. (2023). 2023 Focused Update of the 2021 ESC Guidelines for the Diagnosis and Treatment of Acute and Chronic Heart Failure: Developed by the Task Force for the Diagnosis and Treatment of Acute and Chronic Heart Failure of the European Society of Cardiology (ESC) with the Special Contribution of the Heart Failure Association (HFA) of the ESC. Eur. Heart J..

[8] Marx N., Federici M., Schütt K., Müller-Wieland D., Ajjan R.A., Antunes M.J., Christodorescu R.M., Crawford C., Di Angelantonio E., Eliasson B. (2023). 2023 ESC Guidelines for the Management of Cardiovascular Disease in Patients with Diabetes: Developed by the Task Force on the Management of Cardiovascular Disease in Patients with Diabetes of the European Society of Cardiology (ESC). Eur. Heart J..

[9] McMurray J.J.V., Solomon S.D., Inzucchi S.E., Køber L., Kosiborod M.N., Martinez F.A., Ponikowski P., Sabatine M.S., Anand I.S., Bělohlávek J. (2019). Dapagliflozin in Patients with Heart Failure and Reduced Ejection Fraction. N. Engl. J. Med..

[10] Packer M., Anker S.D., Butler J., Filippatos G., Pocock S.J., Carson P., Januzzi J., Verma S., Tsutsui H., Brueckmann M. (2020). Cardiovascular and Renal Outcomes with Empagliflozin in Heart Failure. N. Engl. J. Med..

[11] Anker S.D., Butler J., Filippatos G., Ferreira J.P., Bocchi E., Böhm M., Brunner-La Rocca H.-P., Choi D.-J., Chopra V., Chuquiure-Valenzuela E. (2021). Empagliflozin in Heart Failure with a Preserved Ejection Fraction. N. Engl. J. Med..

[12] Elrakaybi A., Laubner K., Zhou Q., Hug M.J., Seufert J. (2022). Cardiovascular Protection by SGLT2 Inhibitors—Do Anti-Inflammatory Mechanisms Play a Role?. Mol. Metab..

[13] Lytvyn Y., Bjornstad P., Udell J.A., Lovshin J.A., Cherney D.Z.I. (2017). Sodium Glucose Cotransporter-2 Inhibition in Heart Failure: Potential Mechanisms, Clinical Applications, and Summary of Clinical Trials. Circulation.

[14] Biegus J., Fudim M., Salah H.M., Heerspink H.J.L., Voors A.A., Ponikowski P. (2023). Sodium-Glucose Cotransporter-2 Inhibitors in Heart Failure: Potential Decongestive Mechanisms and Current Clinical Studies. Eur. J. Heart Fail..

[15] Çamcı S., Yılmaz E. (2022). Effects of Sodium-Glucose Co-Transporter-2 Inhibition on Pulmonary Arterial Stiffness and Right Ventricular Function in Heart Failure with Reduced Ejection Fraction. Medicina.

[16] Carluccio E., Biagioli P., Alunni G., Murrone A., Zuchi C., Coiro S., Riccini C., Mengoni A., D’Antonio A., Ambrosio G. (2018). Prognostic Value of Right Ventricular Dysfunction in Heart Failure with Reduced Ejection Fraction: Superiority of Longitudinal Strain over Tricuspid Annular Plane Systolic Excursion. Circ. Cardiovasc. Imaging.

[17] Cinar T., Saylik F., Cicek V., Pay L., Khachatryan A., Alejandro J., Erdem A., Hayiroglu M.I. (2024). Effects of SGLT2 Inhibitors on Right Ventricular Function in Heart Failure Patients: Updated Meta-Analysis of the Current Literature. Kardiol. Pol..

[18] Guazzi M., Naeije R. (2021). Right Heart Phenotype in Heart Failure with Preserved Ejection Fraction. Circ. Heart Fail..

[19] Vijiiac A., Onciul S., Guzu C., Scarlatescu A., Petre I., Zamfir D., Onut R., Deaconu S., Dorobantu M. (2021). Forgotten No More—The Role of Right Ventricular Dysfunction in Heart Failure with Reduced Ejection Fraction: An Echocardiographic Perspective. Diagnostics.

[20] Riccardi M., Pagnesi M., Corso R., Sammartino A.M., Tomasoni D., Inciardi R.M., Lombardi C.M., Adamo M., Nodari S., Metra M. (2024). Prognostic Role of TAPSE to PASP Ratio in Outpatients with Left Ventricular Systolic Dysfunction. ESC Heart Fail..

[21] Wang J., Li X., Jiang J., Luo Z., Tan X., Ren R., Tanimura T.L., Wang M., Zhang C. (2024). Right Ventricular–Pulmonary Arterial Coupling and Outcome in Heart Failure with Preserved Ejection Fraction. Clin. Cardiol..

[22] Antit S., Mrabet A., Fathi M., Fekih R., Boussabeh E., Zakhama L. (2025). Tricuspid Annular Plane Systolic Excursion/Pulmonary Arterial Systolic Pressure Ratio as a Predictor of Outcome in Acute Heart Failure. Tunis. Medicale.

[23] Wu Z., Xie M., Zhang L., He Q., Gao L., Ji M., Lin Y., Li Y. (2025). Prognostic Implication of Right Ventricular–Pulmonary Artery Coupling in Valvular Heart Disease. Front. Cardiovasc. Med..

[24] Palmiero G., Cesaro A., Galiero R., Loffredo G., Caturano A., Vetrano E., Rinaldi L., Salvatore T., Ruggiero R., Di Palo M.R. (2023). Impact of Gliflozins on Cardiac Remodeling in Patients with Type 2 Diabetes and Reduced Ejection Fraction Heart Failure: A Pilot Prospective Study. GLISCAR Study. Diabetes Res. Clin. Pract..

[25] Gamaza-Chulián S., Díaz-Retamino E., González-Testón F., Gaitero J.C., Castillo M.J., Alfaro R., Rodríguez E., González-Caballero E., Martín-Santana A. (2021). Effect of Sodium-Glucose Cotransporter 2 (SGLT2) Inhibitors on Left Ventricular Remodelling and Longitudinal Strain: A Prospective Observational Study. BMC Cardiovasc. Disord..

[26] Zhang N., Wang Y., Tse G., Korantzopoulos P., Letsas K.P., Zhang Q., Li G., Lip G.Y.H., Liu T. (2022). Effect of Sodium-Glucose Cotransporter-2 Inhibitors on Cardiac Remodelling: A Systematic Review and Meta-Analysis. Eur. J. Prev. Cardiol..

[27] Vaduganathan M., Docherty K.F., Claggett B.L., Jhund P.S., de Boer R.A., Hernandez A.F., Inzucchi S.E., Kosiborod M.N., Lamet L.S.P., Martinez F. (2022). SGLT-2 Inhibitors in Patients with Heart Failure: A Comprehensive Meta-Analysis of Five Randomised Controlled Trials. Lancet.

[28] Heidenreich P.A., Bozkurt B., Aguilar D., Allen L.A., Byun J.J., Colvin M.M., Deswal A., Drazner M.H., Dunlay S.M., Evers L.R. (2022). 2022 AHA/ACC/HFSA Guideline for the Management of Heart Failure: A Report of the American College of Cardiology/American Heart Association Joint Committee on Clinical Practice Guidelines. J. Am. Coll. Cardiol..

[29] Abbate M., Fusco F., Scognamiglio G., Merola A., Palma M., Grimaldi N., Barracano R., Borrelli N., Ppaccioli G., Sorice D. (2023). Dapagliflozin in Adults with a Systemic Right Ventricle: Initial Results from the DAPA-SERVE Study. Eur. Heart J..

[30] Amsallem M., Mercier O., Kobayashi Y., Moneghetti K., Haddad F. (2018). Forgotten No More: A Focused Update on the Right Ventricle in Cardiovascular Disease. JACC Heart Fail..

[31] Chemla D., Berthelot E., Assayag P., Attal P., Hervé P. (2018). Physiopathologie Hémodynamique du Ventricule Droit [Pathophysiology of Right Ventricular Hemodynamics]. Rev. Mal. Respir..

[32] Axelsen J.S., Nielsen-Kudsk A.H., Schwab J., Ringgaard S., Nielsen-Kudsk J.E., de Man F.S., Andersen A., Andersen S. (2023). Effects of Empagliflozin on Right Ventricular Adaptation to Pressure Overload. Front. Cardiovasc. Med..

[33] Sanz J., Sánchez-Quintana D., Bossone E., Bogaard H.J., Naeije R. (2019). Anatomy, Function, and Dysfunction of the Right Ventricle: JACC State-of-the-Art Review. J. Am. Coll. Cardiol..

[34] Kusunose K., Seno H., Yamada H., Nishio S., Torii Y., Hirata Y., Saijo Y., Ise T., Yamaguchi K., Fukuda D. (2018). Right Ventricular Function and Beneficial Effects of Cardiac Rehabilitation in Patients with Systolic Chronic Heart Failure. Can. J. Cardiol..

[35] Schmeisser A., Rauwolf T., Groscheck T., Kropf S., Luani B., Tanev I., Hansen M., Meißler S., Steendijk P., Braun-Dullaeus C.R. (2021). Pressure-Volume Loop Validation of TAPSE/PASP for Right Ventricular Arterial Coupling in Heart Failure with Pulmonary Hypertension. Eur. Heart J. Cardiovasc. Imaging.

[36] Rako Z.A., Kremer N., Yogeswaran A., Richter M.J., Tello K. (2023). Adaptive versus Maladaptive Right Ventricular Remodelling. ESC Heart Fail..

[37] Saucedo-Orozco H., Voorrips S.N., Yurista S.R., de Boer R.A., Westenbrink B.D. (2022). SGLT2 Inhibitors and Ketone Metabolism in Heart Failure. J. Lipid Atheroscler..

[38] Lopaschuk G.D., Verma S. (2020). Mechanisms of Cardiovascular Benefits of Sodium Glucose Co-Transporter 2 (SGLT2) Inhibitors: A State-of-the-Art Review. JACC Basic Transl. Sci..

[39] Russo V., Malvezzi Caracciolo D’Aquino M., Caturano A., Scognamiglio G., Pezzullo E., Fabiani D., Del Giudice C., Carbone A., Bottino R., Caso V. (2023). Improvement of Global Longitudinal Strain and Myocardial Work in Type 2 Diabetes Patients on Sodium-Glucose Cotransporter 2 Inhibitors Therapy. J. Cardiovasc. Pharmacol..

[40] Nevola R., Alfano M., Pafundi P.C., Brin C., Gragnano F., Calabrò P., Adinolfi L.E., Rinaldi L., Sasso F.C., Caturano A. (2022). Cardiorenal Impact of SGLT-2 Inhibitors: A Conceptual Revolution in the Management of Type 2 Diabetes, Heart Failure and Chronic Kidney Disease. Rev. Cardiovasc. Med..

[41] Rivera F.B., Tang V.A.S., De Luna D.V., Lerma E.V., Vijayaraghavan K., Kazory A., Shah N.S., Volgman A.S. (2023). Sex Differences in Cardiovascular Outcomes of SGLT-2 Inhibitors in Heart Failure Randomized Controlled Trials: A Systematic Review and Meta-Analysis. Am. Heart J. Plus.

[42] Singh A.K., Singh R. (2020). Gender Difference in Cardiovascular Outcomes with SGLT-2 Inhibitors and GLP-1 Receptor Agonist in Type 2 Diabetes: A Systematic Review and Meta-Analysis of Cardiovascular Outcome Trials. Diabetes Metab. Syndr..

[43] Wittnich C., Tan L., Wallen J., Belanger M. (2013). Sex Differences in Myocardial Metabolism and Cardiac Function: An Emerging Concept. Pflugers Arch..

[44] Butler J., Filippatos G., Siddiqi T.J., Ferreira J.P., Brueckmann M., Bocchi E., Böhm M., Chopra V.K., Giannetti N., Iwata T. (2022). Effects of Empagliflozin in Women and Men with Heart Failure and Preserved Ejection Fraction. Circulation.

[45] Obokata M., Olson T.P., Reddy Y.N.V., Melenovsky V., Kane G.C., Borlaug B.A. (2018). Haemodynamics, Dyspnoea, and Pulmonary Reserve in Heart Failure with Preserved Ejection Fraction. Eur. Heart J..

[46] Reddy Y.N.V., Carter R.E., Sorimachi H., Omar M., Popovic D., Alogna A., Jensen M.D., Borlaug B.A. (2024). Dapagliflozin and Right Ventricular–Pulmonary Vascular Interaction in Heart Failure with Preserved Ejection Fraction: A Secondary Analysis of a Randomized Clinical Trial. JAMA Cardiol..

[47] Kosiborod M.N., Angermann C.E., Collins S.P., Teerlink J.R., Ponikowski P., Biegus J., Comin-Colet J., Ferreira J.P., Mentz J.R., Nassif M.E. (2022). Effects of Empagliflozin on Symptoms, Physical Limitations, and Quality of Life in Patients Hospitalized for Acute Heart Failure: Results from the EMPULSE Trial. Circulation.

[48] Oriecuia C., Tomasoni D., Sala I., Bonfioli G.B., Adamo M., Gussago C., Lombardi C.M., Pagnesi M., Savarese G., Metra M. (2024). Sodium Glucose Co-Transporter 2 Inhibitors and Quality of Life in Patients with Heart Failure: A Comprehensive Systematic Review and Meta-Analysis of Randomized Controlled Trials. Eur. Heart J. Cardiovasc. Pharmacother..

[49] Iatridi F., Carrero J.J., Cornec-Le Gall E., Kanbay M., Luyckx V., Shroff R., Ferro C.J. (2024). KDIGO 2024 Clinical Practice Guideline for the Evaluation and Management of Chronic Kidney Disease. Kidney Int..

[50] Lang R.M., Badano L.P., Mor-Avi V., Afilalo J., Armstrong A., Ernande L., Flachskampf F.A., Foster E., Goldstein S.A., Kuznetsova T. (2015). Recommendations for Cardiac Chamber Quantification by Echocardiography in Adults: An Update from the American Society of Echocardiography and the European Association of Cardiovascular Imaging. J. Am. Soc. Echocardiogr..

[51] Voigt J.U., Pedrizzetti G., Lysyansky P., Marwick T.H., Houle H., Baumann R., Pedri S., Ito Y., Abe Y., Metz S. (2015). Definitions for a Common Standard for 2D Speckle Tracking Echocardiography: Consensus Document of the EACVI/ASE/Industry Task Force to Standardize Deformation Imaging. J. Am. Soc. Echocardiogr..

