# Supplementary material for: Impact of Gliflozins on Right Heart Remodeling in Italian Patients with Type 2 Diabetes and Heart Failure: Results from the GLISCAR Real-World Study
Source: Pharmaceuticals (Basel). 2025 Aug 14;18(8):1200. doi: 10.3390/ph18081200 (PMC12388898; doi:10.3390/ph18081200)

Supplementary Materials

Landmark analysis evaluating whether changes over 12-month follow-up were influenced by age, sex, and BMI for each echocardiogram parameter. The mean age in the male group was 63.23 ± 15.81 years, and it was 63.50 ± 15.37 years in the female group.

Figure S1. Left ventricular end-diastolic diameter (LVEDD).

Sex: p=0.0079; Time p=0.0046; Sex x Time interaction p=0.2459

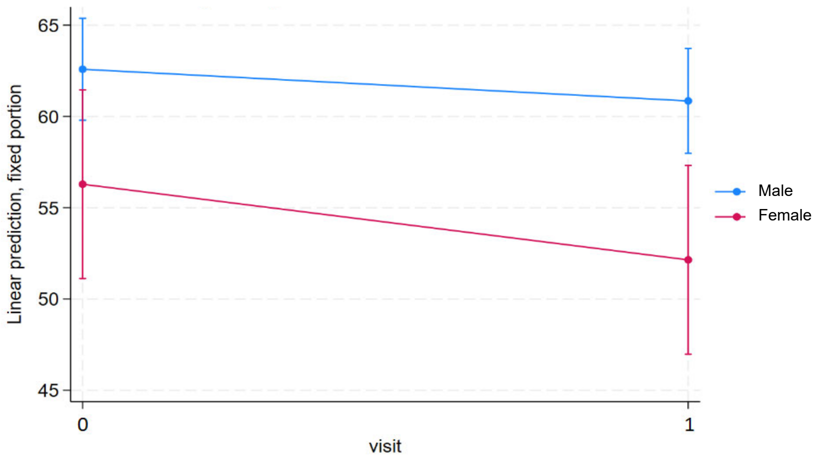

Figure S2. Left ventricular end-systolic diameter (LVESD).

Sex: p=0.0009; Time p=0.0064; Sex x Time interaction p=0.2349

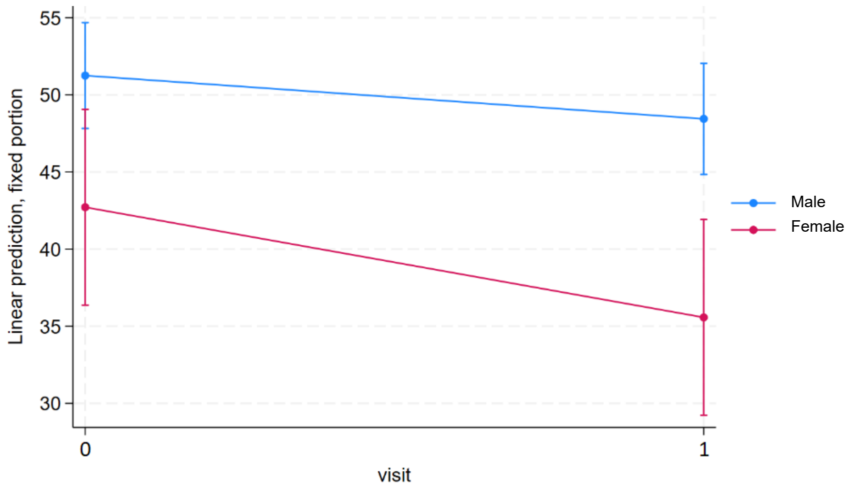

Figure S3. Interventricular septum diameter (IVSD).

Sex: p=0.1116; Time p=0.4017; Sex x Time interaction p=0.7310

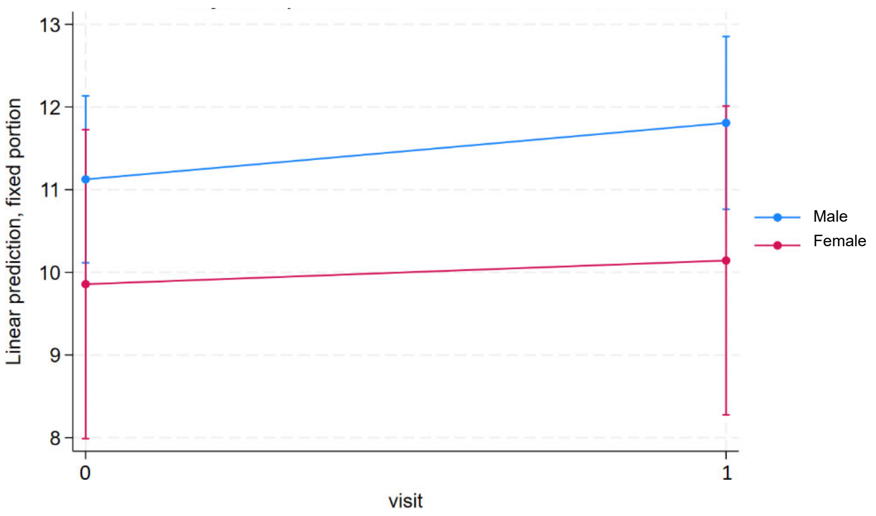

Figure S4. Left ventricular end-diastolic volume (LVEDV).

Sex: p=0.0045; Time p=0.0698; Sex x Time interaction p=0.3791

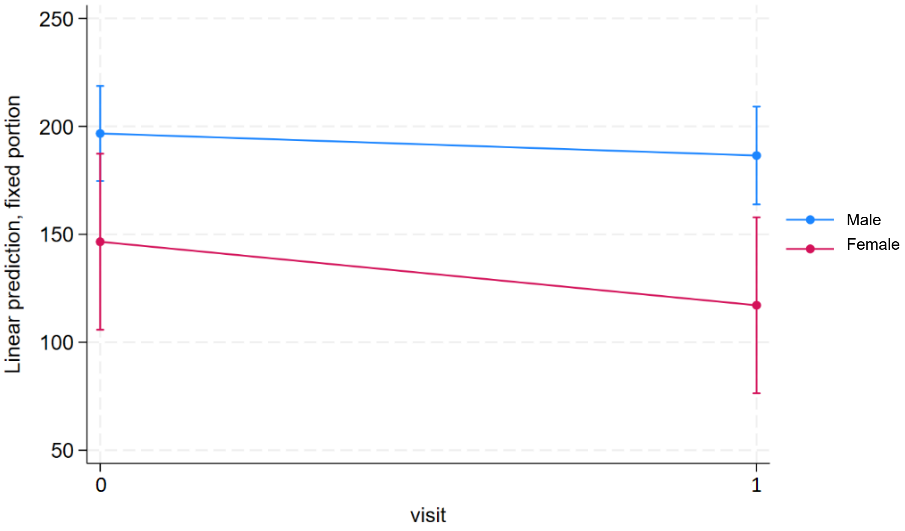

Figure S5. Left ventricular end-systolic volume (LVESV).

Sex: p=0.0044; Time p=0.0051; Sex x Time interaction p=0.3864

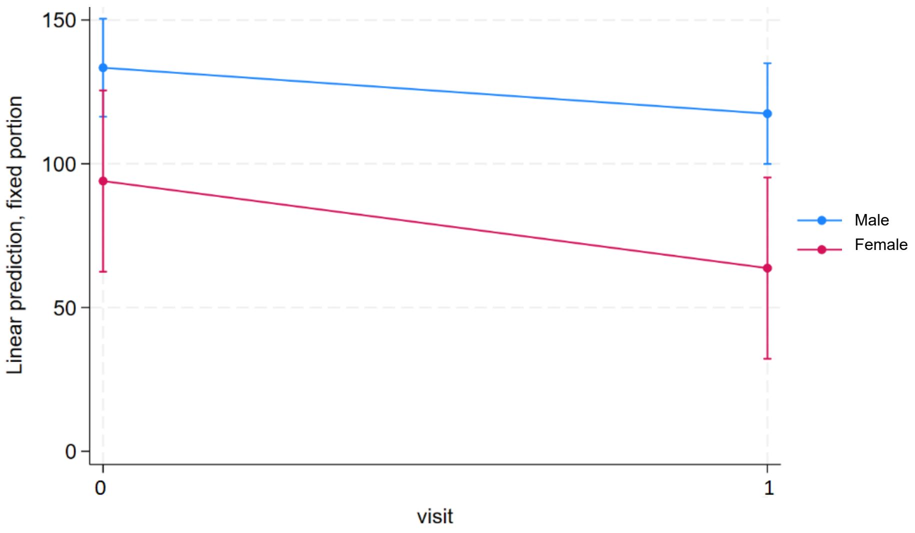

Figure S6. Global longitudinal strain (GLS).

Sex: p=0.0064; Time p=0.0009; Sex x Time interaction p=0.3455

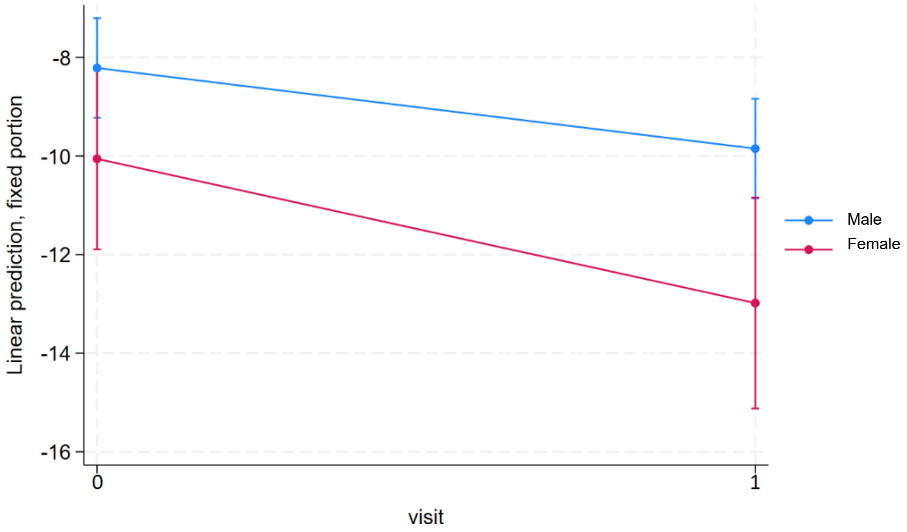

Figure S7. Right atrial area (RAA).

Sex: p=0.0128; Time p=0.0763; Sex x Time interaction p=0.0957

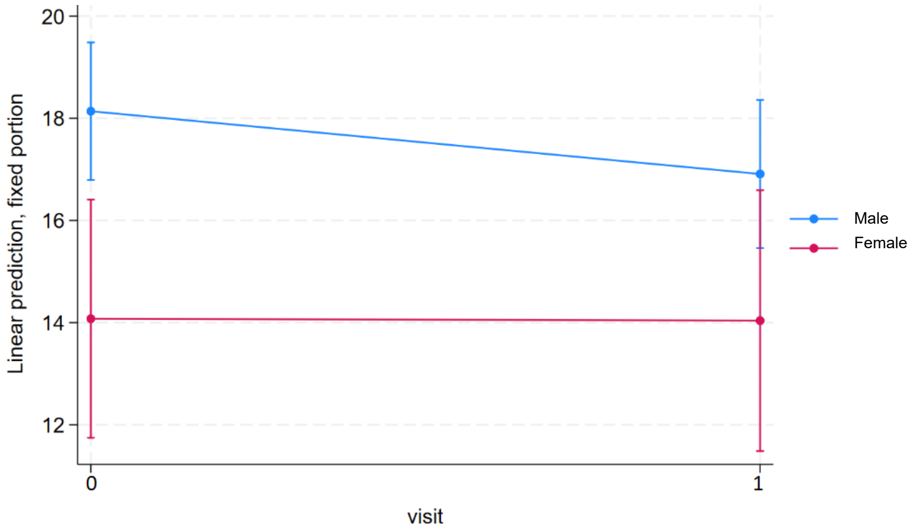

Figure S8. Right atrial emptying index (RAEI).

Sex: p=0.2521; Time p=0.5107; Sex x Time interaction p=0.8497

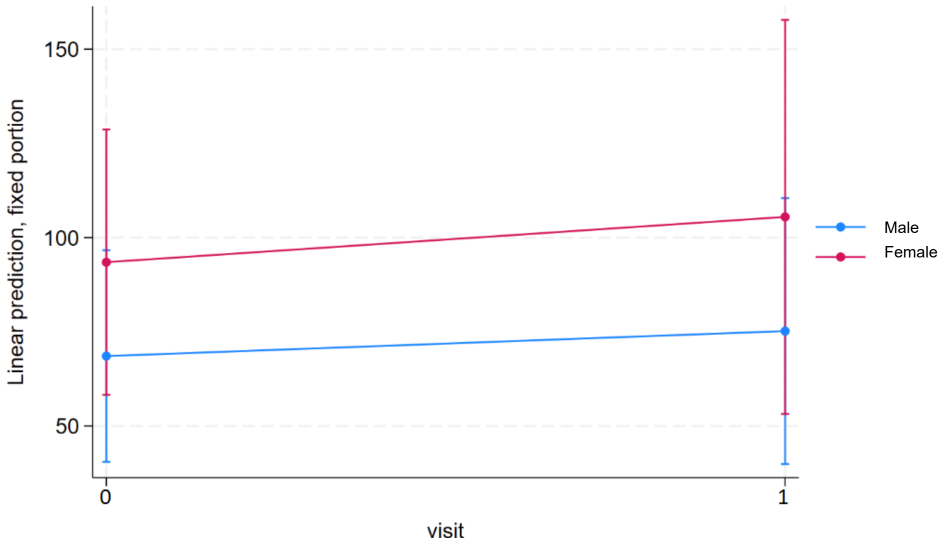

Figure S9. Total right atrium emptying fraction (TRAEF).

Sex: p=0.1309; Time p=0.1300; Sex x Time interaction p=0.8647

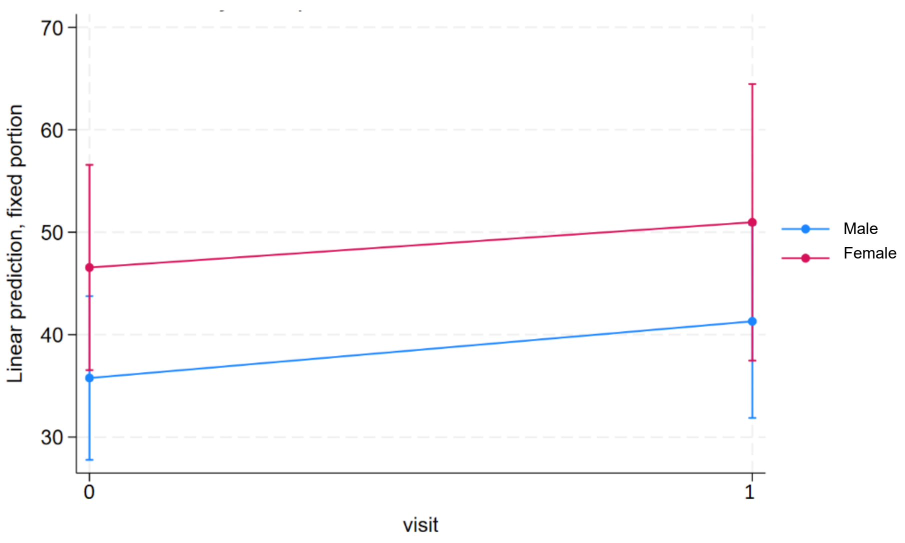

Figure S10. Right ventricular diameter 3 (RVD3).

Sex: p=0.4873; Time p=0.9794; Sex x Time interaction p=0.7052

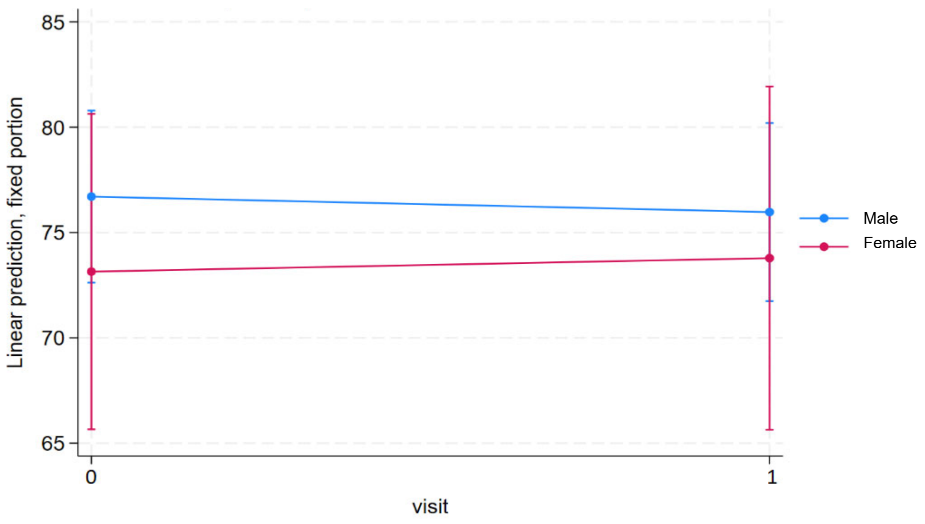

Figure S11. Tricuspid annular plane systolic excursion (TAPSE).

Sex: p=0.2857; Time p=0.0217; Sex x Time interaction p=0.7637

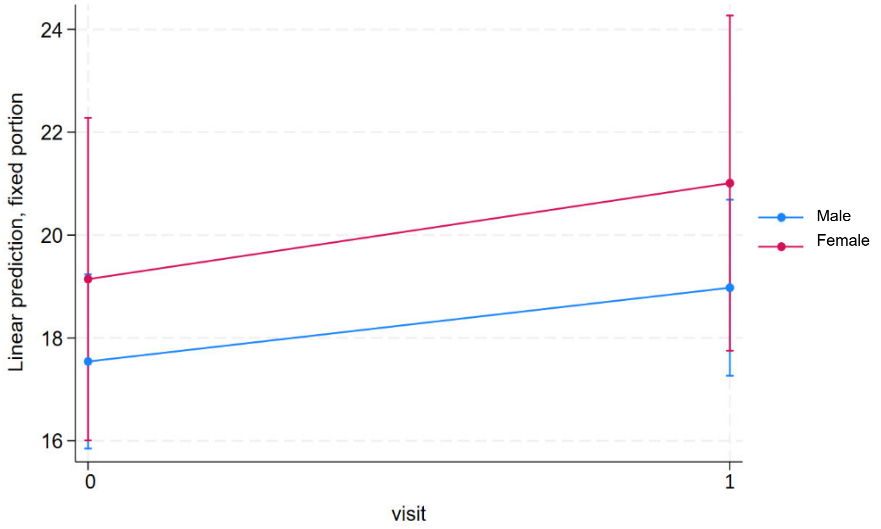

Figure S12. Fractional area shortening (FAC).

Sex: p=0.8271; Time p=0.3663; Sex x Time interaction p=0.7244

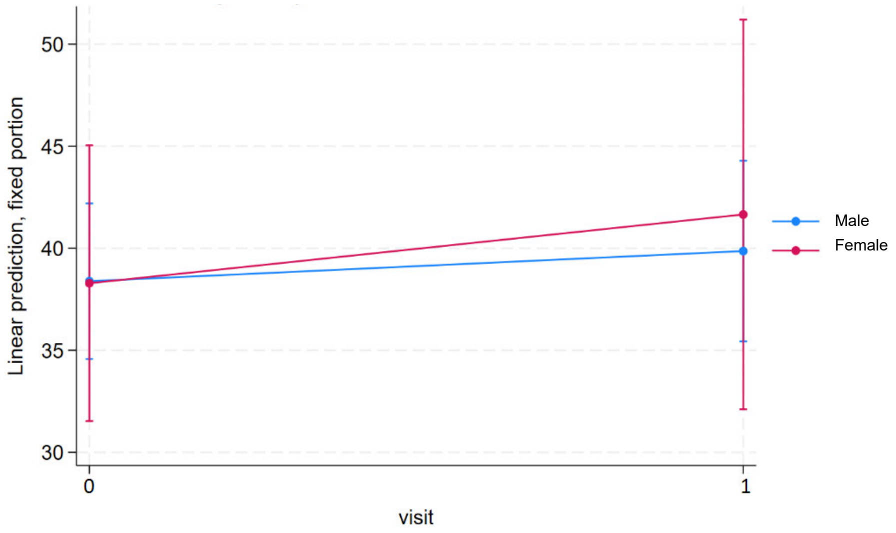

Figure S13. Pulmonary artery systolic pressure (PASP).

Sex: p=0.1427; Time p=0.3509; Sex x Time interaction p=0.3736

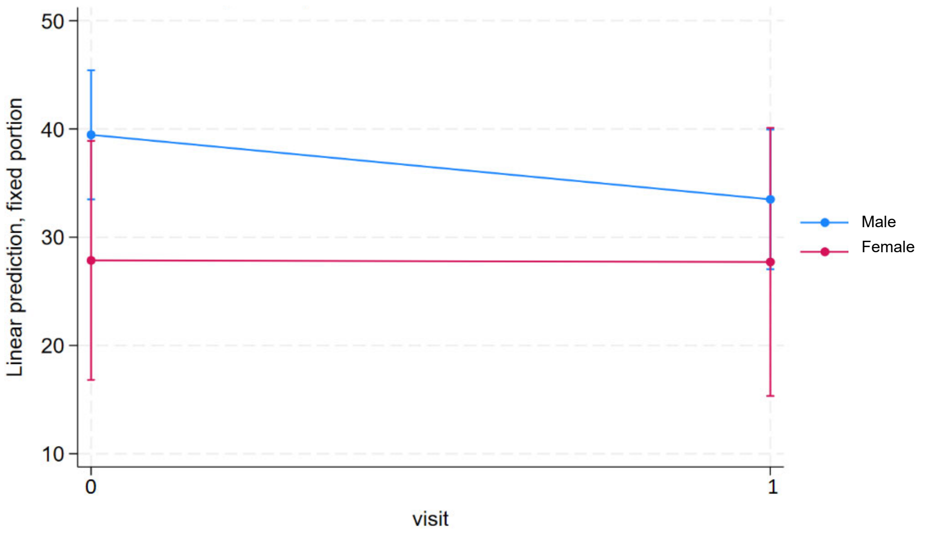

Supplement: Supplementary file 1 [file pharmaceuticals-18-01200-s001.zip › pharmaceuticals-3811835-supplementary.pdf]
