# Supplementary material for: Impact of Gliflozins on Right Heart Remodeling in Italian Patients with Type 2 Diabetes and Heart Failure: Results from the GLISCAR Real-World Study
Source: Pharmaceuticals (Basel). 2025 Aug 14;18(8):1200. doi: 10.3390/ph18081200 (PMC12388898; doi:10.3390/ph18081200)
Supplement: Supplementary file 1 [file pharmaceuticals-18-01200-s001.zip › STROBE_checklist_GLISCAR.pdf]

STROBE Statement—Checklist of items that should be included in reports of ***cohort studies***

	Item No	Recommendation
**Title and abstract**	1	The title clearly identifies the design as a real-world prospective observational cohort study. The abstract summarizes the background, objectives, setting, participants, intervention, main findings (including effect estimates and confidence intervals), and a cautious interpretation emphasizing the exploratory nature of the study.
**Introduction**		
Background/rationale	2	The introduction presents a solid scientific rationale for investigating the effects of SGLT2 inhibitors on right ventricular (RV) remodeling in patients with heart failure with reduced ejection fraction (HFrEF) and type 2 diabetes (T2D). While extensive literature exists on left ventricular outcomes, RV effects remain understudied.
Objectives	3	The primary aim is clearly stated: to assess longitudinal changes in RV function over 12 months. Secondary aims include right atrial function, pulmonary artery pressures, and RV–arterial coupling.
**Methods**		
Study design	4	The manuscript describes a prospective, non-randomized, non-controlled observational study conducted in two tertiary hospitals in Italy.
Setting	5	Recruitment occurred between February 2021 and June 2022, with baseline and 12-month follow-up visits.
Participants	6	Inclusion and exclusion criteria are detailed. Patients had stable HFrEF (LVEF ≤40%) and T2D and were initiating SGLT2i therapy.<br>Exclusion criteria: advanced pulmonary hypertension, severe RV dysfunction, or recent changes in HF therapy.<br>Patients were recruited during routine visits and followed for 12 months with standard care and scheduled echo assessments.
Variables	7	Exposure:<br>Initiation of SGLT2 inhibitor therapy in patients with HFrEF and T2D.<br>Primary outcome:<br>Change in right ventricular function at 12 months, assessed based on TAPSE, RVFAC, and TAPSE/PASP ratio.<br>Secondary outcomes: NYHA functional class, estimated pulmonary artery systolic pressure (PASP), qualitative evaluation of right atrial size and function.<br>Confounders:<br>All patients were clinically stable and maintained unchanged background therapy, minimizing potential confounding.
Data sources/ measurement	8*	**Echocardiographic data** (TAPSE, RVFAC, PASP, TAPSE/PASP ratio, RA size and function) were collected by experienced cardiologists using standardized transthoracic protocols and vendor-specific software. Measurements were performed at baseline and 12 months, following EACVI/ASE guidelines.

		**Clinical data** (NYHA class) were obtained from medical records and confirmed at scheduled visits.
Bias	9	Standardized protocols and consistent medication regimens minimized selection and performance bias.
Study size	10	The sample size (n=31) was based on recruitment feasibility, with justification provided in the limitations.
Quantitative variables	11	Continuous variables were described using appropriate descriptive statistics. Time-based changes were analyzed using paired tests and linear models
Statistical methods	12	The manuscript details the statistical tests used, methods for delta calculation, interaction models, and adjustments for baseline characteristics. Missing data were not an issue due to full follow-up.
**Results**		
Participants	13*	A total of 50 patients were screened for eligibility across the two participating centers. After applying the inclusion and exclusion criteria, 31 patients were enrolled and completed both baseline and 12-month echocardiographic assessments. These individuals were included in the final analysis. Patients were excluded primarily due to severe right ventricular dysfunction, advanced pulmonary hypertension, or clinical instability at the time of screening.<br>Figure 1 illustrates the study's flow chart.
Descriptive data	14*	Baseline demographic and clinical characteristics are summarized in Table 1. These include key demographic variables (age, sex), clinical status indicators (NYHA functional class, systolic and diastolic blood pressure, heart rate), comorbidities (duration of type 2 diabetes, presence of atrial fibrillation, chronic kidney disease), and current pharmacological therapy.<br>Baseline echocardiographic data are also reported, with a specific focus on left ventricular ejection fraction (LVEF), tricuspid annular plane systolic excursion (TAPSE), right ventricular fractional area change (RVFAC), pulmonary artery systolic pressure (PASP), and the TAPSE/PASP ratio.<br>There were no missing data in the study's dataset.
Outcome data	15*	Follow-up echocardiographic and laboratory outcomes are summarized in Tables 2 and 3.

Main results	16	The manuscript reports both unadjusted values and interaction effects (e.g., sex-by-time). Confidence intervals accompany key findings, such as TAPSE and PASP.
Other analyses	17	Subgroup analyses included landmark regression by sex, age, and BMI. A sex-specific response in LVEF improvement was noted.
**Discussion**		
Key results	18	The authors interpret the improvements in RV function and hemodynamics as potentially attributable to SGLT2i therapy.
Limitations	19	Limitations include the small sample size, the absence of a control group, potential measurement variability, and reduced generalizability.
Interpretation	20	The discussion contextualizes the findings within the broader literature and emphasizes the exploratory and hypothesis-generating nature of the study.
Generalizability	21	Results are cautiously interpreted, with recognition that findings may not extend to patients with severe RV dysfunction or different clinical settings.
**Other information**		
Funding	22	**Funding:** The study did not receive external funding.<br>**Ethics and consent:** Ethics approval and informed consent were obtained and reported in the manuscript.
